# Supplementary figures and images for: Molecular Control of Non-Muscle Myosin II-A Aggregation and Intracellular Dynamics by motor- or tail-specific MYH9 Mutations
Source: bioRxiv. 2025 May 21:2025.05.20.654665. Preprint. [Version 1] doi: 10.1101/2025.05.20.654665 (PMC12139846; doi:10.1101/2025.05.20.654665)

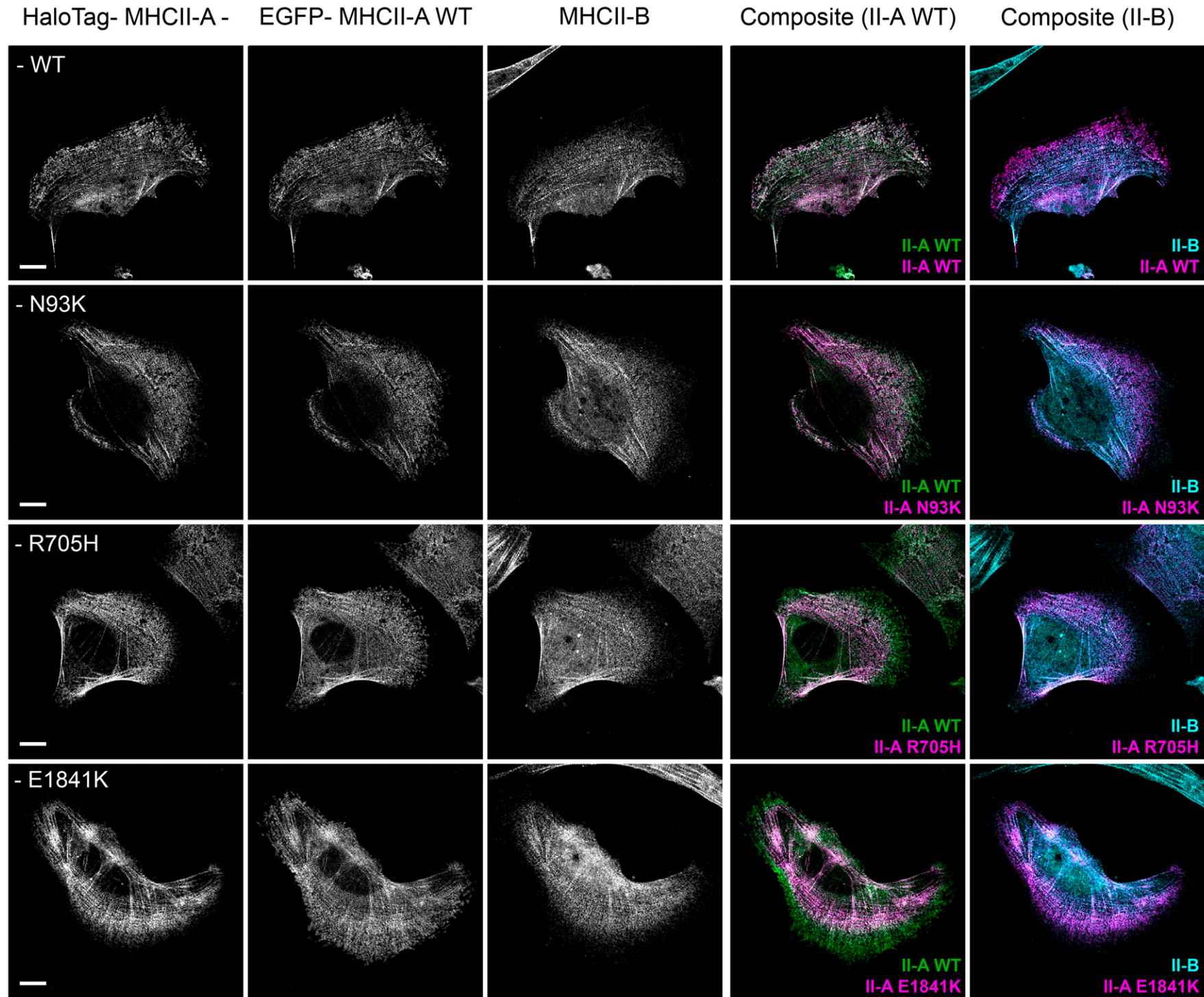

Llorente-González et al., 2025. Fig. S1

**A**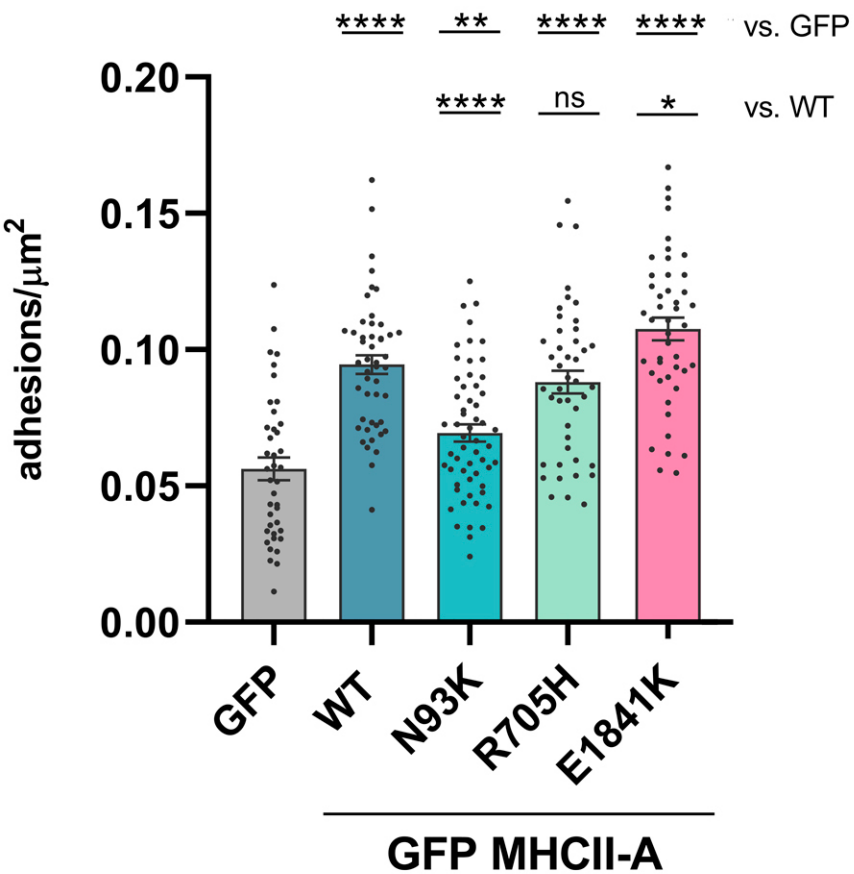**B**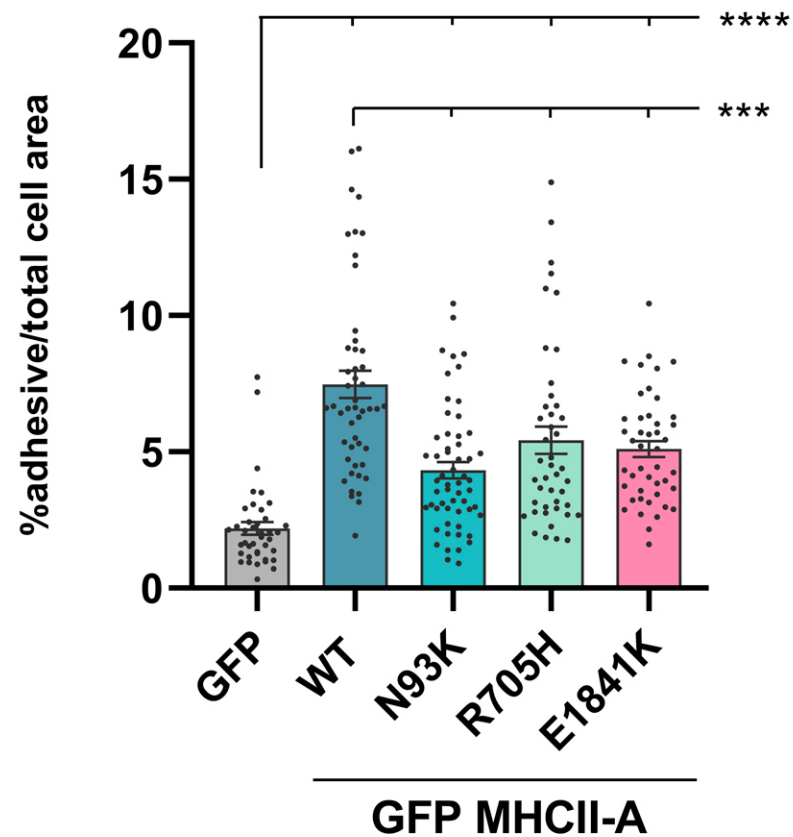

**A**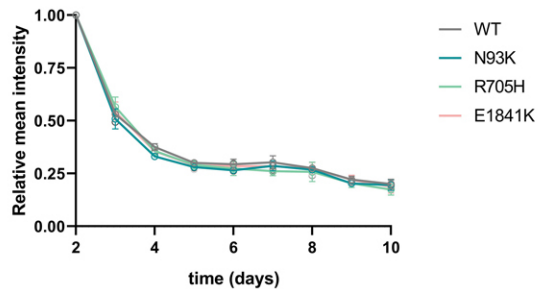**B**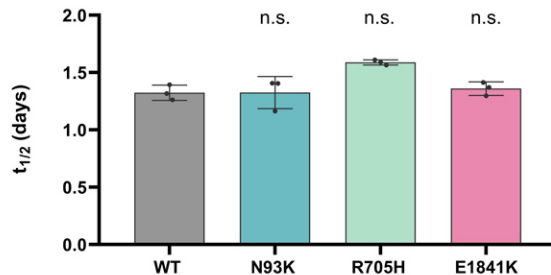**C**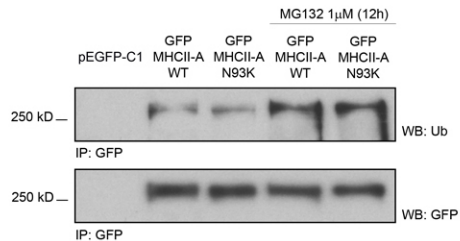

Supplement: 1 — Figure S1. Localization of MHCII-A mutants in U2OS cells. U2OS cells were transfected with Halo-MHCII-A wild type (WT) the GFP-MHCII-A carrying the indicated mutation. After 24h, cells were allowed to spread on fibronectin (2 μg/mL)-coated dishes overnight, fixed and stained for endogenous MHCII-B (magenta in overlay). In the II-A WT composite images, Halo-MHCII-A WT is magenta, and the mutant is green. In the II-B composite images, the mutant is magenta and endogenous MHCII-B is blue. Representative cells are shown. Scale bar=10 μm. Figure S2. NM2-A mutations differentially regulate adhesion assembly and elongation Quantification of the number of adhesions per μm2 (A) and percentage of adhesive area (B) of COS7 cells expressing the indicated GFP-MHCII-A mutant. Adhesion quantification was carried out as indicated for Fig. 2 by staining endogenous paxillin. Data was calculated as indicated in Material and Methods. n>30 cells from two independent experiments. Significance was calculated using non-parametric Mann Whitney test. * p<0.05; ** p<0.01; *** p<0.001; **** p<10−4; n.s., not significant. Figure S3. MYH9-RD or DFNA17 mutations neither affect NM2-A cellular degradation rates nor induce differences in ubiquitination. (A) Relative fluorescence intensity (± SEM) of Halo+ cells from three independent experiments. Mean fluorescence intensity was normalized to initial intensity levels to correct for differences in the expression levels among conditions. No significant differences are observed at any time point. (B) Half-life (± SEM) of Halo-MHCII-A WT or the mutant constructions. Data were adjusted to a one phase exponential decay model. There were no differences in the fluorescence decay rate between WT or the indicated mutants. Statistical significance was evaluated with the non-parametric Mann-Whitney U test. n.s., not significant (vs. WT). (C) Ubiquitin levels of immunoprecipitated EGFP-MHCII-A WT or N93K in COS-7 cells. 48h post-transfection, the heavy chain was [file NIHPP2025.05.20.654665v1-supplement-1.pdf]
